# Viral Vector Vaccines Against ASF: Problems and Prospectives

**DOI:** 10.3389/fvets.2022.830244

**Published:** 2022-03-11

**Authors:** Rustam Kh. Ravilov, Albert A. Rizvanov, Danil N. Mingaleev, Antonina G. Galeeva, Elena Yu. Zakirova, Eduard A. Shuralev, Catrin S. Rutland, Nail I. Khammadov, Marina A. Efimova

**Affiliations:** ^1^Kazan State Academy of Veterinary Medicine named after N. E. Bauman, Kazan, Russia; ^2^Kazan (Volga Region) Federal University, Kazan, Russia; ^3^Federal Center for Toxicological, Radiation and Biological Safety, Kazan, Russia; ^4^Kazan State Medical Academy, Kazan, Russia; ^5^Faculty of Medicine and Health Sciences, School of Veterinary Medicine and Science, University of Nottingham, Nottingham, United Kingdom

**Keywords:** African swine fever, domestic pigs, vaccine, viral vectors, disease control

## Abstract

African swine fever (ASF) is a highly contagious viral disease affecting pigs, with mortality rates a primary focus as they can reach up to 100%. The widespread and colossal economic losses from ASF have impacts on the development of animal husbandry practices in most countries within Africa, Asia, and Europe. Currently, a variety of approaches toward the development of vaccines against ASF are being employed. A promising new concept centered around more economical and time-consuming vaccine production is based on the use of viral vectors to deliver selected immunogens. This review discusses the results obtained from testing various viral vectors as carriers of targeted ASF virus genes. The safety and prospects of viral vectors, the possibilities around modulating cellular and humoral immune responses by choosing genes expressing immunodominant antigens, and the degree of protection in experimental animals from infection with a lethal dose of virulent ASF virus strains have been shown and discussed.

## Introduction

Pig farming is expanding in size all over the world and it is also a strategically significant and important industry for global food security. Nevertheless, this sector of the economy is especially vulnerable due to various transboundary infectious diseases, among which African swine fever (ASF) is currently a serious threat to worldwide pig production (1, 2). African swine fever virus (ASFV) is unique, the only member of the Asfarviridae family, and is the causative agent of one of the most dangerous diseases in the Suidae family (commonly referred to as pigs, hogs, or boars). ASFV has a large double-stranded DNA genome ranging from 170 to 190 kb, which encodes over 150 open reading frames (ORFs), depending on the virus strain, and is the only known DNA to contain arbovirus (3, 4). The ASFV virion has a diameter of ~200 nm, and is a complex multi-shell and multi-icosahedral particle containing at least 68 different structural viral polypeptides and 21 cellular proteins, and has a much more complex structure than previously thought. The exact functions of significant parts of the structural and non-structural proteins within ASFV are presently unknown (5, 6). ASFV encodes new proteins that are involved in modulating the host immune response, the virulence of the virus to the domestic pig, and the ability of the ASFV to replicate and spread in the tick vector. Despite the existence of only one type of virus, at present 24 of its genotypes have been described, with genotypes 23 and 24 described in 2017 and 2018, respectively (7, 8).

ASF affects domestic and wild members of the *Suidae* family, causing a wide variety of symptoms ranging from chronic or persistent infection through to acute hemorrhagic fever, and causes up to 100% mortality (9). Cases of disease in wild boars are also of concern, both for their possibility to spread the disease further and for the consequences relating to biodiversity and nature management (10). Over the past 10 years, ASF has spread over three continents, and as a result, the threat from this transboundary disease now has unprecedented geographical coverage (11, 12). ASF traditionally presents on the African continent, and by 2005 had been registered in 32 different countries throughout the world. In 1978, the disease was introduced to Sardinia, where it became endemic. In 2007, the disease was first confirmed in the Caucasus region—in Georgia, from there the virus gradually spread into neighboring countries (Armenia, Azerbaijan, Russia, and Belarus) via both domestic pigs and wild boars. In the European Union, the first case of ASF was registered in 2014, where, as of the end of 2021, it continues to be registered in 16 countries (13). In August 2018, ASF was registered for the first time in Asia—in China, and since then has affected 16 countries in the region. In 2019-2020, the first occurrence of ASF in Oceania had been reported by Timor-Leste and Papua New Guinea. In 2021, the disease reappeared in America after a 40-year absence—it was introduced to the Dominican Republic and then to Haiti. In total, as of 2020, ASF has been detected in five different regions in the world within 32 countries, has affected more than 1 million pigs and in excess of 28 thousand wild boars, and has caused the loss of more than 1.5 million animals (14).

In global agriculture, one of the acute and most important problems over the past few years has been the development of an effective vaccine against ASFV. Unfortunately, traditional methods have not developed vaccines that provide a wide range of cross-immune responses. Therefore, it is important to take into account more modern technologies when designing and developing vaccines for this disease (15). In this review, viral vector-based vaccines as carriers of key ASFV genes are discussed, their abilities in modulating the desired cellular and humoral immune response are assessed, and their protective potentials have been compared. This will be useful for advancing research pertaining to the improvement of viral vectors for further development of vaccines against ASF that have the potential to be highly protective.

## Viral Vector Vaccines: Advantages and Disadvantages

A promising modern technology is the use of viral vectors as carriers for the delivery of desired immunogens (16). The viral vector concept was introduced by Jackson et al. (17) in 1972, when recombinant DNA was created from the SV40 virus using genetic engineering, much has been discussed around this method in the literature since its inception. Subsequently, in 1982 Moss et al. (18) reported the use of a vaccinia virus as a vector for transient expression of the hepatitis B surface antigen HBsAg. After the initial successful results testing the first vector vaccine on chimpanzees, a wide range of different viruses were used as a basis for creating vaccines based on viral vectors: retroviruses, lentiviruses, adenoviruses, poxviruses, alphaviruses, arenaviruses, herpesviruses, flaviviruses, paramyxoviruses, and rhabdoviruses (19). These viral vectors have been optimized to improve their genome packaging ability, cellular tropisms and replication capabilities in order to tailor the desired immune responses (20).

Viral vector vaccines combine many of the benefits of DNA vaccines and live attenuated vaccines. Like DNA vaccines, viral vector vaccines carry DNA into the host cell to induce antigenic proteinsthat can be matched to stimulate a range of immune responses, including antibodies, T helper cells (CD4+ T cells), and cytotoxic T lymphocytes (CTL, CD8+ T cells) mediated immunity. Vaccines with a viral vector, unlike DNA vaccines, can actively penetrate the cells of immunized animals and replicate as a live attenuated vaccine (21). The specific properties of each vector are determined by the carrier virus, and every vector has its own advantages and disadvantages. The main disadvantages of viral vector vaccines are that they represent a more complex production process (22), they risk genomic integration, development of host-induced neutralizing antibodies to the carrier virus itself can occur, and/or it may not be possible to use the same technology for repeated vaccinations (23, 24). In this regard, innovative strategies have been developed to overcome these shortcomings, some of which include the “incorporating antigen into the capsid” strategy, generating chimeric vectors (25, 26), covalent modifications, and helper-dependent vectors (27, 28).

The safety of viral vectors is ensured by the removal or replacement of the virulence genes from the corresponding viruses with immunogens, or by disrupting the replication of the viral vector. Moreover, viral vectors are inherently compatible for differentiating infected from vaccinated animals (DIVA), i.e., the immunogens encoded by the viral vector can serve as vaccine markers. To date, only a few studies have been conducted to assess the immunogenicity and protective efficacy of candidate vaccines with the ASF genes carried by viral vectors, but only a few of them have been tested against virulent infection with the ASFV (29) (Table 1).

**Table 1:** Known viral vectors used in the development of an ASF vaccine or as a carrier of ASF genes fragments.

**Gene/protein**	**Vector**	**Challenge strain**	**Outcome**	**Citation**
**Baculovirus vectors**				
Protein p12	Acpl2 recombinant baculovirus	E70	Pigs immunized with purified recombinant Acpl2 did not develop protective immunity against African swine fever	Carrascosa et al. (30)
HA	HABv recombinant baculovirus	E75	Pigs immunized with the recombinant HABv were protected from lethal infection, they developed antibodies that inhibited HA and neutralized antibodies specific to the 75 kDa structural protein	Ruiz-Gonzalvo et al. (31)
Proteins p54, p30	Bacp54, Bacp30 with [35S]Met/Cys recombinant baculoviruses	E75L8	Pigs immunized with the recombinant p54 or p30 proteins elicited neutralizing antibodies that inhibited viral attachment. The immunized pigs were not protected from the lethal infection	Gómez-Puertas et al. (32)
Chimeric p54/30 Proteins	Bac54/30 recombinant baculovirus	E75	Pigs immunized with the chimeric protein survived after the lethal infection with the virulent ASFV. The immunized pigs exhibited showed neutralizing antibodies and an approximately two log reduction in maximum viremia titers compared to control pigs	Barderas et al. (33)
Proteins p30, p54, p72, and p22	pBlueBac III recombinant baculovirus	Pr4 isolate	Pigs immunized with the recombinant proteins developed neutralizing antibodies to the p30, p54, p72 and p22 proteins from of the ASF virus, but they were not protected against infection with the pathogenic strain	Neilan et al. (34)
Proteins p54, p30, secretory hemagglutinin sHA	BacMam-sHAPQ based on baculovirus vector	E75	4 out of 6 immunized pigs remained free from ASFV following after infection with the homologous virus, 2 pigs showed viremia titers similar to control animals. The levels of specific antibodies observed after ASFV experimental infection in sera from pigs immunized with BacMam-sHAPQ were indistinguishable from those found in control pigs	Argilaguet et al. (35)
**Pox vaccinia virus vectors**				
Genes B646L (p72), E183L (p54), EP153L (C-type lectin), EP402R (CD2v), O61R (p12)	Modified vaccinia virus Ankara (MVA)	Not confirmed by experimental infection	Immunization with the selected antigens induced specific antibodies and a T-cell immune response following primary immunization of pigs	Lopera-Madrid et al. (36)
Genes B646L, EP153R, EP402R (CD2v)	MVA-ASFV recombinant construction	Not confirmed by experimental infection	Induction of a T-cell response against each of the antigens, but antigen-specific antibodies were not detected in the immunized pigs	Lopera-Madrid et al. (36)
Proteins p30 (RP- 30), p54 (RP-54), pHA-72 (RP-sHA- p72)	Alphavirus replicon particles (RPs)	Not confirmed by experimental infection	Alphavirus-expressed immunogenic proteins ASFV p30, p54, and p72 were tested as prime antigens in the attenuated live vaccine candidate virus prime booster approach, OURT88/3. A correlation was found between protein expression *in vitro* and immunogenicity *in vivo*. Antibodies induced by RP-30 alone were insufficient to neutralize viral infection *in vitro*, compared to infection with OURT88/3, which expresses additional viral neutralizing antigens such as p54 and p72	Murgia et al. (37)
**Adenovirus vectors**				
Proteins p32, p54, pp62, p72	pAd/CMV/V5- DEST	Not confirmed by experimental infection	The multi-antigen Ad-ASFV was immunogenic and safe when given as a primary booster vaccination. It was found that a After primary vaccination there was is a rapid production of antibodies that recognized cells infected with the ASFV and the generation of antigen- specific IFN-γ and antigen-specific CTL responses were generated to all of four ASFV antigens	Lokhandwala et al. (38)
Genes A151R, B119L, B602L, EP402RΔPRR, B438L, K205R, A104R	pAd/CMV/V5- DEST	Not confirmed by experimental infection	Evaluation of the local response and systemic immunity to the introduction of a mixture of recombinant adenoviruses after priming and post-boosting in immunized animals showed that the immunogen was well tolerated and no serious negative effects were observed. The new cocktail of ASFV antigens with the AdV vector was able to safely induce high levels of antibodies and IFN-γ + cellular responses in pigs	Lokhandwala et al. (39)
Genes A151R, B119L, B602L, EP402RΔPRR, B438L, K205R- A104R, pp62, p72	Ad-ASFV + BioMize adjuvant	Georgia 2007/1	Induction of a high level of IgG was observed, but after infection the vaccinated pigs were sick in a more severe form compared to the controls	Lokhandwala et al. (40)
Proteins p32, p54, pp62, p72, p37-34- 14, p150-I, p150-II	Ad-ASFV + BioMize adjuvant	Georgia 2007/1	Induction of stronger humoral immunity was noted, but 8/10 of the vaccinated and 4/5 pigs in the control group died of the disease or reached the experimental endpoint 17 days after infection	Lokhandwala et al. (40)
	Ad-ASFV + ZTS-01 Adjuvant		Induction of weaker antibody responses was observed, but 4/9 of the vaccinated pigs died of the disease, while 5 survivors showed low clinical scores and no viremia during 17 days after challenge, whereaswhile 4/5 of the control animals died of the disease or reached the experimental endpoint	
Genes I215R, I73R, CP530R (pp62), CP204L (p32), MGF110-5L, B646L (p72), MGF110-4L, M448R, L8L, E146L, C129R, A151R, MGF110-1L, L10L, K78R, E184L, E165R, CP312R	rAd + MVA	OUR T88/1	Proteins have been identified that can induce ASFV-specific cellular and humoral immune responses in pigs. Pools of viral vectors expressing these genes did not protect animals from severe disease, but did reduce viremia in a proportion of pigs following ASFV challenge	Netherton et al. (41)
Genes B602L, B646L, CP204L, E183L, E199L, EP153R, F317L, MGF505-5R	rAd5 + MVA	OUR T88/1	Immunization with this pool of antigens protected 100% of pigs from lethal disease after infection with a usually lethal dose of virulent ASFV	Goatley et al. (42)
Protein p72	rNDV (??. MG7)	E70	Mice immunized with rNDV/p72 developed high titers of IgG antibodies specific to p72 fromof ASFV and had higher levels of IgG1 than IgG2a. Immunization also caused T-cell proliferation and the secretion of IFN-γ and IL-4	Chen et al. (43)
Gene DP71L	pHR-SIN- CSGW	Malawi Lil-20/1, Benin 97/1	The DP71L gene did not increase the levels of eIF2α phosphorylation *in vitro*, which indicateds that DP71L was is not the only factor required by the virus to control the levels of eIF2α phosphorylation during infection	Zhang et al. (44)
Gene I329L	pHR-CMV- eGFP	Not confirmed by experimental infection	Outcomes indicated that ORF I329L can disrupt TLR3-controlled cellular responses that result in both IFN-β production and NFκB activation	De Oliveira et al. (45)
Proteins *UBCv1*, UBCv1mut	pLVX-Puro	Not confirmed by experimental infection	The results suggested that ASF UBCv1 manipulates the innate immune response directed toward the NF-κB and AP-1 pathways	Barrado-Gil et al. (46)
Genes SV40LT, pTERT	pLVSIN-EF1α neo	Experimental infection is not expected	The ability of a new cell line of immortalized porcine kidney macrophages (IPKM) against ASF infection was examined. It was summarized that IPKM can be a valuable tool for the isolation, replication and genetic manipulation of ASF in both basic and applied research	Masujin et al. (47)

## Baculovirus Vectors

The baculovirus BacMam was first used as a vector for the delivery of targeted genes to create diagnostic drugs and vaccines against ASF. Baculoviruses are double-stranded enveloped DNA viruses belonging to the *Baculoviridae* family, it are also one of the most studied insect viruses. The most widely used baculovirus for basic virology and biotechnology is *Autographa californica* multiple nucleopolyhedrovirus (AcMNPV). There are a wide range of mammalian cells capable of baculovirus transduction, with their non-toxic and non-replicative nature, large packaging capacity, and their ease of production make baculoviruses promising tools for gene therapy. The main advantage of the baculovirus is the safety of its insect cell expression system, as such baculoviruses are considered non-pathogenic for humans, and the range of baculovirus hosts is limited to insects and invertebrates (48). BacMams, modified baculoviruses, contain mammalian expression cassettes for gene delivery and expression in mammalian cells. The BacMam system combines the advantages of transient viral expression, ease of generation, and broad cellular tropism. This allows fast, simple and flexible gene overexpression experiments in various mammalian cell lines (49).

Carrascosa et al. (50) expressed the p12 protein using a baculovirus vector to immunize domestic pigs. The recombinant p12 protein, in a dose dependent manner, was able to suppress the production of ASFV in pig macrophages infected with a number of different viral isolates, including attenuated, virulent, highly passaged in tissue culture, and non-hemadsorbing strains. This indicated the fundamental role of p12 in the early interactions of the virus with receptors of natural target cells. However, pigs immunized with purified recombinant p12 did not develop protective immunity against ASF (30, 50, 51).

Ruiz-Gonzalvo et al. (31) constructed a recombinant baculovirus carrying the ASFV hemagglutinin (HA) gene, homologous to the CD2 surface antigen on T-lymphocytes. ASFV hemagglutinin, expressed by baculovirus, a viral protein, has been used to immunize domestic pigs against ASF. Pigs immunized with recombinant HA developed hemagglutination inhibiting antibodies and virus neutralizing antibodies that recognized the 75 kDa structural protein, and were therefore protected from a lethal infection. The vaccinated animals were able to develop a protective response similar to that obtained during experiments investigating passive transfer of antibodies carried out in convalescent animals (30, 51, 52).

Likewise, Gómez-Puertas et al. (32) used baculovirus to express p54 and p30 proteins, which are involved in virus attachment and antibody-mediated defense. Immunization of pigs with the recombinant p54 or p30 proteins elicited neutralizing antibodies that inhibited viral attachment or internalization, respectively. However, the immunized pigs were not protected from the lethal infection, and the course of the disease in these animals remained unaltered. On the contrary, immunization with a combination of p54 and p30 proteins simultaneously stimulated both mechanisms of virus neutralization and dramatically altered the course of the disease. This provided various degrees of protection, ranging from delayed onset of the disease through to complete protection against viral infection.

In subsequent studies, immunization with baculovirus-expressing p54/p30 fusion protein reduced viremia and protected all pigs from virulent challenges with the ASFV strain E75 (33). At the same time, pigs immunized, with a mixture containing p30 + p54 + p72 + p22 proteins expressed by baculovirus, were not protected from homologous infection with the virulent Pr4 strain of the ASF virus genotype I (34). The results from this study indicated that the neutralizing antibodies elicited by these proteins were insufficient for protection.

Argilaguet et al. (35) showed that immunization of pigs with BacMam-sHAPQ, a baculovirus-based construct encoding p30, p54 and secretory hemagglutinin or sHA, induced antigen-specific T-cell responses in pigs, and thus protected pigs from a sublethal infection in the absence of specific antibodies. After the challenge, 4/6 of the immunized pigs, excluding negative controls, were virus free. These results demonstrated the key role of T cells in protecting against ASFV.

Baculoviruses have also been used to express the highly antigenic ASF virus proteins virus p54 and p30, encoded by the E183L and CP204L genes, respectively, for diagnostic purposes. Comparative analysis of the sequences of these genes from different strains of field viruses isolated in different geographical regions collected in different years showed that both genes were completely conserved among the isolates. Comparative analyzes showed that p54 elicited the best reactivity in western blotting, and p30 was most prominent via ELISA (53). Heimerman et al. (54) also demonstrated the effectiveness of the baculovirus vector for the production of highly specific monoclonal antibodies (mAbs) against the recombinant antigenic fragment of the p72 protein. Monoclonal antibodies recognize conserved regions and can increase the sensitivity of antigen and antibody-based assays, enabling detection of isolates belonging to a wide range of genotypes.

## Pox Vaccinia Virus Vectors

Modified vaccinia virus Ankara (MVA) is currently being used as a recombinant viral vector for the development of vaccines against infectious diseases and cancer. MVA can encode one or more foreign antigens and can therefore function as a multivalent vaccine. The vector can be used at biosafety level I, has inherent adjuvant properties, and elicits both humoral and cellular immune responses. The safety and efficacy of vaccines using the MVA vector has been proven by a large number of clinical trials (55). Lopera-Madrid et al. (36) selected potentially new ASFV antigen targets and a delivery system for ASFV recombinant antigens—mammalian cells (HEK 293) and a modified vaccinia virus Ankara (MVA) using the Vaxign *in silico* antigen prediction program. Vaccination with HEK proteins (p72, p54, p12) purified from ASFV induced more humoral immune responses and a less intense cellular immunity. Recombinant modified vaccinia virus MVA-ASFV (B646L, EP153R and EP402R (CD2v) induced T-cell responses against each of the antigens, especially against p72. Although antigen specific antibodies were not induced, T-cell responses to each of the antigens were detected in the immunized pigs. Since robust T-cell and B-cell immune responses to ASFV can consistently be obtained in immunized pigs using this strategy and approach, a primary booster regimen of the ASFV antigen complex could be used in future studies to assess the efficacy of potential vaccines against ASFV. This potentially provides us with the ability to induce stable protective immunity against virulent homologous and heterologous ASFV infections. Despite the fact that the protectiveness of cellular immunity has not been confirmed by infection with the virulent ASF virus, the results obtained were encouraging and suggest that the MVA vector may be a suitable platform for developing vaccines (36, 56).

The reported partial protection outcomes described in previous publications suggests that multiple immune mechanisms are involved in the induction of protection against ASF. In order to provide complete protection, it is likely that both the previously identified potential protective antigens, and those whose role remains to be determined, should be considered. The combination of several antigens, optimal delivery methods and adjuvant systems associated with different immunization strategies may be necessary in order to achieve successful induction of humoral and cell-mediated immunity and to provide complete protection.

## Alphavirus Vectors

Alphavirus vector vaccines have been found to be safe and effective against several viral diseases in pigs, including ASF. Alphaviruses are RNA positive single-stranded viruses belonging to the *Togaviridae* family, they replicate in the cytoplasm of infected cells, thus eliminating concerns about the possible integration of viral genes into the host genome. Known alphaviruses that are currently being evaluated as potential vaccine delivery platforms are the Venezuelan Equine Encephalitis Virus (VEEV), Sindbis Virus (SINV), Semliki Forest Virus (SFV), and VEEV-SINV. The immunogenicity and protective efficacy of alphavirus replicon particle vaccines against parainfluenza virus type 3 (PIV3) and HIV have already been established. Alpha-virus replicon vector technology continues to be used as a platform for vaccines aimed at a variety of viral, bacterial, protozoan, and tumor antigens (57). An alphavirus vector platform was used to deliver replicon particles (RP) expressing ASFV antigens to pigs. Expression of the ASF antigens p30 (RP-30), p54 (RP-54) and pHA-72 (RP-sHA-p72) were tested in Vero cells and for immunogenicity in pigs. RP-30 exhibited the highest expression in Vero cells and was measured as the most immunogenic in pigs, followed by RP-54 then RP-sHA-p72. Pigs primed with two doses of the RP-30 construct were then boosted with a naturally attenuated ASFV isolate OURT88/3. P30 mapping revealed an immunodominant region within amino acid residues 111-130. However, the main effect of the prime boost was to enhance recognition of the epitope covered by the peptide sequence 61-110. The authors revealed a correlation between protein expression *in vitro* and immunogenicity *in vivo*, which makes it possible to predict that RP constructs are most likely immunogenic (37, 56).

Although assessing the abilities of RP-30 + OURT88/3 in relation to protecting against virulent ASFV infection was beyond the scope of this study, the results from this work lay the foundation for future efforts toward developing an effective vaccine which could provide cross-protection against ASF. More research is therefore required to understand the effects of this primary boosting approach on T cell responses and to determine the functional role of the proteins themselves.

## Adenovirus Vectors

The most commonly used viral vectors are vectors constructed from recombinant adenoviruses. Adenoviruses are one of the most genetically diverse DNA viruses and cause non-life-threatening infections of the eye, respiratory or gastrointestinal epithelium in a wide variety of animal and human species. Adenoviruses are excellent vectors for delivering genes or vaccine antigens to target host tissues, and are currently being tested in several vaccine and gene therapy studies. Adenovirus-based vectors have several advantages over other viral vectors, including a wide range of tissue tropism, a well-characterized genome, ease of genetic manipulation, including the capacity of large transgenic DNA inserts, inherent adjuvant properties, the ability to induce resistant transgen-specific T-cells and antibody production, non-replicative nature in the host, and ease of production on a large scale (58). Lokhandwala et al. (38, 39) evaluated the immunogenicity and safety of two ASFV multi-antigen cocktails with live vectors in two independent studies. The first study showed that immunization of pigs with a mixture of adenoviruses expressing structural antigens (p32, p54, pp62, and p72) induced resistant IgG, IFN-γ + T cells, and CTL responses. A second study showed similar results in pigs immunized with a mixture of adenoviruses expressing new antigens, namely A151R, B119 L, B602 L, EP402RΔPRR, B438 L, K205R, and A104R (39). Further research by Lokhandwala et al. (40) evaluated the protective efficacy of both variants of the antigen mixture by intranasal infection of pigs with the ASF Georgia 2007/1 virus. Composition of nine Ad-ASFV antigens (AdA151R, AdB119 L, AdB602 L, AdEP402RΔPRR, AdB438 L, AdK205R-A104R, Adpp62, and Adp72), prepared with BioMize adjuvant, induced a high level of IgG, but when challenged, vaccinated pigs fell ill compared to controls. The composition of seven Ad-ASFV-II antigens (Adp32, Adp54, Adpp62, Adp72, Adp37-34-14, Adp150-I, and Adp150-II) was evaluated using two adjuvants: BioMize and ZTS-01. A mixture of antigens with the BioMize adjuvant induced a higher level of antibodies, but 8 of the 10 vaccinated and 4 of the 5 control experimental pigs became ill, and died or reached the experimental endpoint 17 days after infection. In contrast, the composition of antigens with adjuvant ZTS-01 resulted in lower levels of antibodies, and while 4 of 9 pigs died from the disease, the 5 survivors showed mild clinical signs and no viremia 17 days after infection, whereas 4 of 5 of the control animals died of illness or reached the experimental endpoint. The post-boost data suggested that the primary and low-dose booster groups had the highest recall responses. Based on this finding, during future immunogenicity and efficacy studies it may be useful to test whether low-dose priming and high-dose boosting induce better immune responses. This is especially interesting given that those vaccinated with the Ad-vector with a mixture of II-ZTS antigens showed lower levels of antibodies and had higher survival rates, but despite this clinical disease was not prevented.

A pool of genes ASFV I215R, I73R, CP530R (pp62), CP204L (p32), MGF110-5L, B646L (p72), MGF110-4L, M448R, L8L, E146L, C129R, A151R, MGF110-1LR, L10L, L10L, E184L, E165R, and CP312R driven by replication-deficient primary human adenovirus 5 (rAd) and enhanced by modified vaccinia Ankara (MVA) led to a decrease in clinical signs and a decrease in viremia levels in some pigs after infection with the virulent isolate OUR T88/1 (29, 41).

Goatley et al. (42) identified eight ASF virus genes B602L, B646L, CP204L, E183L, E199L, EP153R, F317L, and MGF505-5R, which, when delivered to pigs using human adenovirus 5 (rAd) and Ankara recombinant modified vaccine, protected 100% of the pigs from fatal disease after infection with a lethal dose of the virulent ASF virus.

It has been shown that adenovirus-based vectors, as well as those based on alphavirus and pox vaccinia virus, induce more effective antigen-specific immune responses compared to other viral vectors. However, despite initially promising immunogenicity data, these vaccines showed limited protection following challenges with virulent ASF, and in some cases this resulted in increased disease outcomes. It was also found that prime immunization with an adenoviral vector and booster immunization with a vector based on the Ankara vaccinia virus could protect pigs from a lethal disease, which may become the basis for further development of a subunit vaccine against this devastating disease.

## Lentivirus Vectors

Since the early 2000s, lentiviral vectors have been considered as reliable and safe tools for stable gene transfer into eukaryotic cells (59). Unlike other vectors derived from oncoretroviruses, they provide stable gene delivery into most non-dividing primary cells, express the transgene for a long period of time, and exhibit low immunogenicity. Reports of the use of lentivectors (LV) in ASFV genetic engineering are very limited. There is information regarding the expression in the LV system of an early viral protein, ubiquitin-conjugating enzyme (UBCv1), which is the only known conjugating enzyme encoded by a virus that modulates the transmission of signals of innate immunity and inflammation (46), and plays an important role in the formation of antiviral reactions in the host organism. In particular, in this study, LVs were used to overexpress UBCv1. A study by De Oliveira et al. (45) demonstrated the possibility of expression in LV systems of the ASFV I329L gene, a previously uncharacterized gene encoding a glycosylated protein expressed in the cell membrane and on its surface, inhibiting dsRNA-stimulated activation of NFκB and IRF3, two key players of innate immunity. According to the authors, the deletion of the I329L gene provides further rational strategy for creating a vaccine with an attenuated deletion mutant. Zhang et al. (44) presented successful attempts at expressing the ASFV DP71L protein in LV systems; demonstrating that DP71L caused dephosphorylation of eukaryotic translation initiation factor 2 alpha (eIF2α) in resting cells and acted to enhance the expression of cotransfected reporter genes.

Masujin et al. (47) used modified LVs to introduce genes of large T-antigen SV40 (SV40LT) and porcine telomerase reverse transcriptase (pTERT) genes into primary porcine kidney macrophages. A new immortalized cell line was obtained with characteristics showing intermediate and late macrophage phenotypes, which demonstrated competence against ASFV infection and suitability for a wide range of *in vitro* ASFV assays.

## Newcastle Disease Virus Vectors

There is a single report on a mouse model evaluation of a recombinant Newcastle disease virus expressing the p72 protein. This viral vector was shown to be safe and immunogenic (43). However, it is difficult to predict how these results would apply to pigs. This highlights the importance of evaluating vaccine prototypes using the target animal species, pigs.

## Conclusions and Future Studies

ASFV is an important cross-border virus that continues to spread across Europe and Asia and poses a threat to the global pig industry and food security (13). Prevention and control of ASF is very difficult due to the lack of available vaccines and effective therapeutic measures. ASFV is able to interfere with various cellular signaling pathways, leading to immunomodulation, which makes the development of an effective vaccine extremely challenging (60, 61).

Known strategies for the development of vaccines against ASF can be subdivided into broad categories such as: live attenuated ASF virus, inactivated ASF virus, live vector subunit, mammalian expression plasmid and their combinations (62, 63). All of these approaches have limitations that impede rapid progress in the development of safe and effective vaccines which can combat the virus.

Viral vectors represent an attractive broadly applicable platform for vaccine development due to their abilities in inducing stable antigen-specific humoral and cellular immune responses, eliminating the risk of reversion to a virulent state, meeting safety requirements, and ensuring effective differentiation between infected and vaccinated animals. In addition, viral vectors are capable of accommodating large inserts into their genome, thus providing a flexible platform for antigen construction. Vectors based on baculovirus, adenovirus, alphavirus, and pox vaccinia virus have been shown to induce effective antigen-specific ASFV responses (7, 36, 38, 43, 45, 50, 56, 64). However, despite promising reports on immunogenicity, these vaccination regimens have shown limited protection following challenges with the virulent ASFV and, in some cases, have resulted in disease progression.

The development of an ASF vaccine is largely impeded by gaps in our knowledge about the virus and the complex host-virus interactions involved in infection and immunity. To develop an effective vaccine, fundamental omics research is urgently needed to elucidate the functions of ASFV genes, to identify protective proteins and their combinations as vaccine targets, and possible delivery systems that provide stable antigen-specific humoral and cellular immune responses. It is still largely unknown which targets of ASFV play important roles in virulence, immunopathology, or protection. In addition, it is very important to ascertain the right balance between mediated antibodies and cells that provide immune responses toward ASFV. Immune hyperstimulation appears to be a key factor in the course of ASFV disease, of particular significance is the evidence indicating that high antibody levels appear to be particularly detrimental with respect to clinical outcome and protection. A shift in focus toward fewer immunogenic ASFV antigens and the concomitant identification of new neutralizing antigens or ASFV epitopes may also be helpful. Determining which type of antibody aggravates the course of the disease, and work toward identification the neutralizing epitopes of individual antigens, may also be useful for developing a more targeted immune response, and, as a consequence, strengthen the defense system. At the moment, there is no universal “ideal” vector, and various studies require the use of certain vector systems. All viral vector systems will have their advantages and disadvantages depending on the target cells chosen and the specifics of each study. In particular, the advantages of recombinant adeno-associated vectors (the ability to integrate the target gene into the host genome in the right place, which prevents unwanted mutations; integration into both dividing and resting cells; a wide transduction profile; low immune response; strong and stable expression of the transgene) are isolated them among other viral vectors and make these vectors a popular and versatile tool for gene delivery *in vitro* and *in vivo*.

When choosing a suitable viral vector, one should take into account its safety, ability to transduce both dividing and non-dividing cells, capacity and immunogenicity (65).

The fundamental information presented in this review evaluates the key gene expression efficiencies, both *in vitro* and *in vivo*, based on a variety of viral vectors. Furthermore, the review has highlighted previous failures and mistakes. The combination of this knowledge will help develop the future promising viral vectors, designed to create effective vaccines against ASF.

## Author Contributions

RR: conceptualization and administration of the project, addition of important data, raising funds, revision, and editing of the manuscript. ME, AG, and ES: analysis of the literature, writing, and preparation of the initial manuscript draft. EZ and NK: revision and editing of the manuscript. DM: observations within the manuscript. AR and CR: revision of the manuscript and corrections of results interpretations. All authors approved this manuscript for publication.

## Funding

This work was supported by the state assignments for applied scientific research of the Ministry of Agriculture of the Russian Federation No. 121091400103-2 and by the Kazan Federal University Strategic Academic Leadership Program (PRIORITY-2030). The funders had no role in study design, data collection and analysis, decision to publish, or manuscript preparation.

## Conflict of Interest

The authors declare that the research was conducted in the absence of any commercial or financial relationships that could be construed as a potential conflict of interest.

## Publisher's Note

All claims expressed in this article are solely those of the authors and do not necessarily represent those of their affiliated organizations, or those of the publisher, the editors and the reviewers. Any product that may be evaluated in this article, or claim that may be made by its manufacturer, is not guaranteed or endorsed by the publisher.
